# What does Success Look Like for Leaders of Integrated Health and Social Care Systems? a Realist Review

**DOI:** 10.5334/ijic.5936

**Published:** 2021-11-26

**Authors:** Sarah Sims, Simon Fletcher, Sally Brearley, Fiona Ross, Jill Manthorpe, Ruth Harris

**Affiliations:** 1 King’s College London, UK; 2 Kingston University and St George’s University London, UK

**Keywords:** integrated care, leadership, integration, health, social care

## Abstract

**Introduction:**

Health and social care services in England are moving towards greater integration, yet little is known about how leadership of integrated care teams and systems can be supported and improved. This realist review explores what works about the leadership of integrated care teams and systems, for whom, in what circumstances and why.

**Methods:**

A realist synthesis approach was undertaken in 2020 to explore English language literature on the leadership of integrated care teams and systems, complemented by ongoing stakeholder consultation.

**Results:**

Evidence was identified for seven potentially important components of leadership in integrated care teams and systems: ‘inspiring intent to work together’; ‘creating the conditions’; ‘balancing multiple perspectives’; ‘working with power’; ‘taking a wider view’; ‘a commitment to learning and development’ and ‘clarifying complexity’.

**Discussion:**

Research into the leadership of integrated care teams and systems is limited, with ideas often reverting to existing framings of leadership, where teams and organisations are less complex. Research also often focuses on the importance of who the leader is rather than what they do.

**Conclusion:**

This review has generated new perspectives on the leadership of integrated care teams and systems that can be built upon, developed, and tested further.

## Introduction

Health and social care services in England are moving towards integrated models. NHS policy documents, such as the Five Year Forward View [1] and the more recent NHS Long Term Plan [2] and Integrated Care – Next Steps [3] have emphasised this shift, with the strategic intention of building place-based partnerships drawing on existing cross-sector interdependence between the NHS, social care, local authorities, communities and employers. This legislative intention has been confirmed in the recent White Paper, Integration and Innovation: working together to improve health and social care for all [4]. To ensure success in existing collaborations and integrated systems, a range of development needs has been identified, including leadership [5].

There are many differing definitions of leadership, but there is generally a consensus that leadership involves the direction of a group towards shared goals, wider organisational values, vision and objectives, and the management of ongoing change [6,7]. Effective leadership is claimed to be a key element of well-coordinated and safe health and social care [8,9,10,11] and where leadership is ineffective or absent, this has been linked to reports of failures in care leading to patient/service user harm [12]. Yet despite the frequent rhetoric about the importance of leadership in the success of organisations, much research is based upon small-scale studies in specific contexts or grounded in the dated premise that leaders provide guidance for single or uniprofessional teams [13,14], which overlooks the complexity and inevitable tensions of leading integrated care teams and systems. These leaders do not influence just one organisation or professional group, but instead often work between several organisations, across primary and secondary care, health and social care, publicly funded services, the not-for-profit sector, and private businesses. Leaders of integrated care teams and systems therefore require different skills than their predecessors [15,16], yet there is currently little understanding of what these may be, what the mechanisms for effective leadership across integrated care teams and systems might be, the contexts that might influence it or the nature of the resulting outcomes [17]. We responded to this gap in knowledge by establishing what is already known. We undertook a realist review on the leadership of integrated care teams and systems; comprehensively mapping the evidence base and applying realist principles in the interpretation of the literature to identify the key characteristics which comprise effective leadership practices. This paper reports on the findings of this realist review, exploring what aspects of leadership of integrated care teams and systems work, for whom and in what circumstances (*https://www.journalslibrary.nihr.ac.uk/programmes/hsdr/180106/#/*).

## Methods

The research question for the review was ‘*What aspects of leadership of integrated teams and systems in health and social care work, for whom and in what circumstances?*’ The objectives of the review were:

1. To investigate who are the leaders of integrated care teams and systems and what activities contribute to their leadership roles and responsibilities.
2. To explore how leaders lead/manage integrated care teams and systems that span multiple organisations, agencies and sectors.
3. To develop realist programme theories that explain successful leadership of integrated care teams and systems iteratively through stakeholder consultation and evidence review.
4. To identify the development needs of the leaders of integrated care teams and systems.
5. To provide recommendations about optimal organisational and interorganisational structures and processes that support effective leadership of the integrated care teams and systems.

A realist synthesis methodology [18,19,20,21] was adopted to enable the identification of the key contextual characteristics and mechanisms which contribute to effective leadership practice in integrated care [22]. This methodology involved developing and iteratively refining initial programme theories through both evidence review and stakeholder consultation. Following realist synthesis methodology [18], two distinct search phases were undertaken for this review: Stage 1 and Stage 2. However, due to the complexities in identifying relevant literature for this synthesis, Stage 1 was further expanded into Stages 1a and 1b. It is important to connect and interrogate the process of review to the lived experiences of diverse stakeholders engaged in leadership from different perspectives, therefore we held three stakeholder consultation meetings at relevant time-points. For this purpose, we defined stakeholders as those with real-time experience of leading or working within integrated care teams and systems, patients/service users and carers receiving care from integrated care, opinion leaders and researchers with expertise in the field. Nineteen stakeholders agreed to participate in the group and their comments and observations have been articulated throughout this paper.

### Stage 1

In consultation with Information Services Specialists, the following search strategy was developed: “Integrat*” OR “multi-team*” OR “multiteam*” OR “cross-bound*” OR “cross bound*” OR “cross-organisation*” OR “cross organisation*” OR “cross-sector*” OR “cross sector*” AND “leader*” (Limiter: English language only, where available) and run in the following databases: Embase, HMIC, Social Policy and Practice, CINAHL, Medline, International Bibliography of Social Sciences, PsychInfo and Education Research Complete.

A total of 1,446 empirical research papers was identified, of which 532 duplicate papers were removed, leaving a total of 914 papers for review. These papers were divided between two reviewers (SS and SF), who read each abstract to determine whether it was relevant to the focus of the review (i.e. any team/system which spanned health and social care organisations). A total of 66 potentially relevant papers were then divided between the two reviewers and read in full, after which 23 papers were included. Grey literature relating to policy and organisational-based material was also sought by searching Google, Google Scholar, government and other specialist websites (e.g., NHS Leadership Academy, Skills for Care, The King’s Fund, Advance HE, The Institute of Healthcare Management, Social Care Online, NHS England and NHS Improvement). Key words adapted from the main search strategy were used and included “leader”, “leadership”, “integrated care” and “integrated system”. Enormous numbers of evidence sources were identified but most were not relevant. Forty-one pieces of potentially relevant grey literature were read in full by one reviewer (RH), after which 14 articles were included.

These 37 included papers were then divided between three reviewers (SS, SF and RH), who each independently compiled a list of mechanisms identified within them. The reviewers then discussed and compared their findings, agreeing upon the following preliminary mechanisms:

1. Supportive relationships and trust
2. Team working/collaborative working
3. Shared mission/vision/approach/purpose
4. Shared responsibility/ownership
5. Learning, development and innovation
6. Communication
7. Providing clarity
8. Balancing needs
9. Advocacy
10. External liaison/consensus building

These were reviewed at the first stakeholder consultation meeting, where stakeholders were asked which mechanisms they viewed most pertinent, any lacking relevance and any missed. Stakeholders considered some of the mechanisms relevant to integrated care teams and systems (e.g., ‘providing clarity’), but others (such as ‘communication’ and ‘supportive relationships and trust’) too general and already identified within the generic leadership literature. They considered the review needed greater interrogation to identify the components of leadership that were specific to integrated care teams and systems. This included searching outside the health and social care literature to ascertain whether other fields had identified potentially useful theories around leading integrated teams. Furthermore, a potential mechanism felt to be missing from the synthesis was the use of power dynamics within teams and the way leaders negotiated these. It was suggested that ‘use of power’ therefore be included as a preliminary mechanism. Following this useful feedback, a subsequent search stage (‘Stage 1b’) was undertaken.

### Stage 1b

We returned to previously excluded papers (because they were not based within health and social care) and searched their reference lists for any newly identified papers. Twelve relevant papers were identified and divided between the same researchers for review, who again discussed and compared their mechanisms, including how Stage 1b mechanisms compared to those identified in Stage 1a. We then consulted an international advisor to the study and expert on multi-team systems (MTS), who advised us that integrated care services could be conceived of as MTSs. A search was undertaken on Google Scholar for ‘Leadership of multiteam systems’ and 20 potentially relevant papers were identified and read in full. Fourteen papers were included and divided between two reviewers (SS and SF), who added their findings into the preliminary mechanism descriptions. These descriptions were then emailed to the stakeholder group for review before being presented in full at the second stakeholder consultation meeting. By the end of this meeting, stakeholders concurred that the following mechanisms were pertinent to leading integrated care teams and systems (for a short description of these mechanisms, see ***Table 1***):

1. Inspiring intent to work together
2. Taking a wider view
3. Creating the conditions to work together
4. Clarifying complexity
5. Planning and coordinating
6. Balancing multiple perspectives
7. Working with power
8. Commitment to learning and development
9. Fostering resilience
10. Adaptability of leadership style

**Table 1:** Definitions of the final mechanisms at Stage 1b.


MECHANISM	DESCRIPTION

**Inspiring intent to work together**	Integrated care teams and systems have no statutory basis but depend upon *voluntary* collaboration between NHS and local authority leaders to develop a shared, system-wide approach to strategy, planning and commissioning, financial and performance management. Leaders are effective as advocates for integrated care and for *inspiring intent to collaborate* with staff across the system and outside it, at various levels. They have a supportive management style that promotes team cohesion, trust, respect, reciprocity and collaboration. Not only do leaders champion these values in their own conduct but they also promote them in their staff. They empower and inspire participation from all professionals, use ‘public narratives’ where appropriate and prevent resistance behaviours, ensuring that key values such as cooperation, openness and fairness are instilled into the fabric of the service.

**Taking a wider view**	Integrated services involve cross-boundary working with a wide and varied group of organisations and people with a plurality of interests, goals, aspirations and values. Leaders of integrated teams and systems have experience and insight into the motivations and challenges of other organisations and focus on the bigger picture by acknowledging the importance of making strategic connections with leaders in other parts of the system. They use this knowledge to engage with other leaders, be convincing/persuasive in their communications with others, work through challenges in partnership with other organisations bridging language, thought, world, and goal differences that may otherwise prove detrimental, to come up with collective solutions and to look beyond reactive problem solving by taking a longer-term strategic view. Their political astuteness is a necessary and beneficial set of skills that enable them to get things done for constructive ends. Consequently, the goals of the team are more likely to be achieved. However, political astuteness can also be used to pursue personal or sectional interests.

**Creating the conditions to work together**	Different organisations, teams and individuals bring their own organisational, sectional or professional interests, ways of working and cultures. Leaders of integrated teams understand, are committed to and champion a *shared philosophy, shared mental models* and a *common mission/vision/purpose* for integrated services. Leadership is fundamentally more about participation and collectively creating a sense of direction than it is about control and exercising authority. They provide a clear narrative and direction for their team members to enable and encourage them to align their goals, have a shared focus and to engage in integrated working, rather than think about their own clinical teams, organisations or personal needs. They offer team members a sense of common ownership of the team and its reputation, are willing to delegate responsibilities and provide their colleagues with shared responsibility/accountability for financial, cost and quality targets. As a consequence, role defensiveness or ‘turf wars’ are limited, decision making is assisted, and effort becomes more focussed during times of conflict and disagreement.

**Clarifying complexity**	Many complex and challenging conditions are associated with integrated working, with unclear boundaries, structures and processes, different governance procedures and funding streams but leaders can navigate the tension between certainty and uncertainty and translate this to their teams and/or systems. Leaders employ sensemaking strategies, in which they use a set of available artefacts in order to make the understanding of their message clear and internalised. They are successfully able to negotiate the narrow parameters between oversimplification and exclusionary detail, enabling team members to understand the complexity of disparate policy drivers, legislation, performance requirements, regulatory systems and funding mechanisms to ease working arrangements for the team. They do this by developing policies and initiatives that are easily communicated and understood, with documents explaining how decisions are made and who has the authority to make them. This prevents confusion and enables team members to navigate organisations with multiple decision-making bodies.

**Planning and coordinating**	Leaders coordinate, strategize and serve as a liaison and boundary spanner between their team and the other teams in the system. They actively plan and synchronise the teams within the system, aiding the teams with their timing and executions of plans and helping them to organise intrateam processes with inter-team processes and decision making. When component teams struggle to perform their tasks due to high workloads, leaders can provide backup behaviours by prompting other component teams to provide help, shifting workloads to other teams or proactively offering to help with specific tasks. They employ smooth coordination processes that provide the necessary capacity to the whole system to move nimbly and synchronously. This strategizing and coordination improves both team processes and system performance. However, system leaders must also be mindful of changing and competing demands and be able to switch quickly from the routine to the non-routine. Thus, leaders of systems devote time to ensuring system flexibility. If unexpected changes occur and contingency plans no longer seem appropriate, leaders decide whether to reconsider, abandon or adjust the original plan.

**Balancing multiple perspectives**	There is historic power imbalance between health and social care (e.g., between care homes and the NHS) and between professional disciplines. Leaders ensure balance between the organisational cultures, social mission and business aims of the organisations due to having several specialist areas of knowledge and a good understanding of a broad range of topics. They are enthusiastic ‘change agents’ and demonstrate full, visible and sustained support for service integration. They advocate for those organisations that need greater power and are willing to have difficult conversations with colleagues across different organisations and specialisms and to deal with the uncertainty and ambiguity inherent in complex adaptive systems. This enables greater collaborative and equal working across organisations. Leaders are also able to create balance between professional hierarchies within the team and manage conflict between team members appropriately, working with, and negotiating with, many different stakeholders who have divergent values, goals, ideologies and interests. Leaders recognise tension and work through it with staff in order to develop a condition in which it is safe to challenge, and discussion becomes healthy. A productive balance between harmony and healthy debate is maintained and a coalition is created, with a degree of actionable shared purpose.

**Working with power**	Leaders have an awareness of power dynamics and know that the appropriate use of power within and across teams and organisations can be critical during times of uncertainty. Leaders are aware that power dynamics should be skilfully and intelligently negotiated and recognise that colleagues in other parts of the system are sometimes in a better position to lead on certain initiatives than themselves. In such circumstances, they are willing to shift power, migrate authority and relinquish control where appropriate, i.e. if better outcomes can be achieved. When leaders are unwilling to relinquish control, progress can stall. Leaders step aside, showing interest but not interference and steering. They are also aware that tactics for reducing resistance to change based on threats, manipulation, or misinformation are likely to backfire. Leaders use referent power to bring their teams together (i.e., a charisma that makes others feel comfortable in their presence). This leads to higher team satisfaction during the process of change. Because referent power generally takes time to develop, this finding may highlight the importance of placing individuals who are known, liked, and respected by employees in transition-related positions.

**Commitment to learning and development**	Leaders have a strategic commitment to access external support and rapid learning with other like-minded systems. They are committed to reflecting upon and personally learning from a variety of sources, through formal and informal networks, and to act as a role model for team members, encouraging them to also learn and improve. Leaders establish communities of practice for team learning and the pooling of knowledge. Whilst managers apply proven solutions to known problems, leaders are exposed to situations in which groups need to learn their way out of problems that could not have been predicted. Leaders recognise that training initiatives can increase component team members’ awareness and understanding of their knowledge structures, as well as their ability to regulate then improve the effective coordination of the whole system under dynamic circumstances. They have an interest in innovation and creativity, inviting feedback and embracing change and evidence-based practice for continuous improvement. They encourage team members to generate ideas and explore possibilities but also have a tolerance for things not working and learn how to fail ‘well’.

**Fostering resilience**	Those providing public services need to deal with increased demand, higher expectations from the public about service standards, hostility and psychological projections from the public and the media, often in the context of declining resources for public services. The pace can be relentless and the physical, intellectual, and emotional demands very high. Successful leaders of integrated systems have both the personality and learned skills that foster high resilience, perseverance, and an awareness of the importance of remaining empathic to the public whilst also resilient in terms of their own wellbeing. They put in place social support systems (both within and outside work) and attend appropriate training and personal development programmes to strengthen resilience. Leader stress is therefore reduced.

**Adaptability of leadership style**	Leading an integrated team or system is difficult, given the complexities of moulding two or more organisations into one and the sense of loss or uncertainty that employees may experience as part of this. Collaborative leaders are able to adapt their actions based on the circumstances they confront. They acknowledge particular situations call for particular leadership skills and behaviours. Leaders align their styles according to the situation at hand, combining or switching approaches as necessary, changing strategy towards flexibility and the use of their tacit knowledge. This generates cooperation, cohesiveness and improved communication amongst group members.


### Stage 2

Once the preliminary mechanisms were formulated, a second stage search was undertaken seeking any empirical evidence of these mechanisms. The following search strategy was used: “Integrat*” OR “multi-team*” OR “multiteam*” OR “cross-bound*” OR “cross bound*” OR “cross-organisation*” OR “cross organisation*” OR “cross-sector*” OR “cross sector*” OR “Interorganisation*” OR “Inter-organisation*” AND “leader*” AND “Health” [Limiter: English language only]. This second search was undertaken in the same databases as Stage 1a, with the addition of Social Care Online, Scopus, and PubMed. Handsearching of key journals (e.g., International Journal of Integrated Care, Journal of Interprofessional Care and Journal of Integrated Care) was also undertaken by searching for the term ‘leadership’ in the online access of each journal.

The abstracts of 5,673 papers were read and 420 were deemed to be potentially relevant. These were added to 11 papers which were forwarded by the stakeholder consultation group and study co-applicants; two papers that were picked up in the Stage 1 searches but not in Stage 2; the 14 MTS papers identified in the Stage 1 search; 11 papers identified through searching the reference lists of relevant papers (n = 458). One originally excluded paper plus three papers identified by searching the Nuffield Trust website were also added, resulting in 462 possible papers, although 16 of these were inaccessible through library resources. This left 446 papers, which were divided between two reviewers (SS and SF) and read in full. Thirty-six of these papers were included. A data extraction form was created and completed for each paper read. Each paper was read carefully many times to identify evidence that supported, weakened, modified, or refocused understanding of each mechanism, the contexts that supported them or not and the outcomes associated with it [20]. In line with realist synthesis methodology, conventional approaches to quality appraisal were not used. Rather, each study’s ‘fitness for purpose’ was assessed by considering its relevance and rigour. We then re-contacted the stakeholder group to inform them of the number of included papers and they recommended stopping any further searching of the literature as the process had been comprehensive.

**Figure 1:**
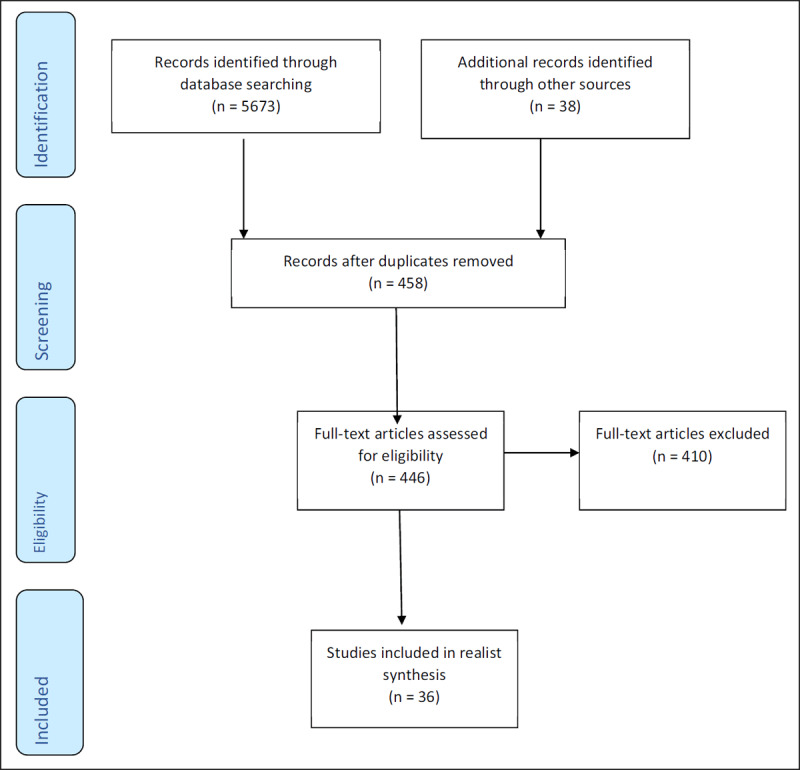
Flowchart of Stage 2 searches.

The evidence collected from these 36 papers was synthesised by drawing together all information on contexts, mechanisms and outcomes and comparing similarities and differences to build a comprehensive description of each mechanism and its role in the leadership of integrated care teams and systems (all 36 papers and the mechanisms identified within them are highlighted in ***Table 2***). This was a long iterative process and discussed in great detail by the research team. These descriptions were emailed to the stakeholder consultation group for review and then discussed in detail at the third, final stakeholder meeting. Due to restrictions in place following the Covid-19 pandemic, a planned face-to-face stakeholder meeting was replaced by online video conferencing software, ‘Teams’, and stakeholders were presented with the evidence found for each mechanism and asked to help develop these descriptions further, using their own lived, research or practice experiences. A flow diagram of the stage 2 search process is provided in ***Figure 1***.

**Table 2:** Papers informing the realist synthesis at Stage 2.


PAPER	INSPIRING INTENT TO WORK TOGETHER	CREATING THE CONDITIONS TO WORK TOGETHER	TAKING A WIDER VIEW	COMMITMENT TO LEARNING AND DEVELOPMENT	CLARIFYING COMPLEXITY	BALANCING MULTIPLE PERSPECTIVES	WORKING WITH POWER	FOSTERING RESILIENCE	GENERAL CONTEXTS	GENERAL OUTCOMES

Aitken (2014)	✓	✓	✓	✓	✓	✓			✓	

Alexander (2001)	✓	✓	✓	✓			✓			

Asakawa (2017)	✓	✓				✓				

Atkinson (2002)	✓		✓		✓	✓	✓			

Axelsson (2009)	✓	✓	✓			✓	✓			

Balasubramanian (2012)		✓								

Benzer (2015)		✓				✓				

Best (2017)	✓	✓				✓	✓			

Brouselle (2010)	✓									

Carroll (2015)		✓		✓						

Choi (2012)	✓	✓			✓	✓	✓			

Chreim (2010)	✓	✓	✓	✓	✓	✓	✓		✓	

Cohen (2006)		✓	✓	✓			✓			

Cramm (2012)	✓					✓	✓			

Dayan (2019)						✓	✓			

Dickinson (2007)	✓	✓	✓	✓			✓			

Grenier (2011)			✓	✓	✓	✓	✓			

Karam (2017)						✓	✓			

Kharicha (2005)						✓				

Klinga (2016)		✓		✓	✓				✓	✓

Ling (2012)	✓	✓	✓	✓	✓					✓

Lunts (2012)	✓		✓			✓			✓	

Nicholson (2018)	✓	✓		✓	✓	✓				

Payne (2019)	✓									

Rees (2004)				✓	✓	✓				

Roberts (2018)									✓	

Rosen (2011)	✓	✓				✓				

Scragg (2006)	✓	✓	✓							

Shand (2019)									✓	

Shaw (2011)	✓	✓		✓					✓	

Stuart (2012)	✓					✓	✓			

Touati (2006)							✓			

Van Eyk (2002)		✓								

Williams (2012a)	✓	✓	✓	✓		✓	✓			

Williams (2012b)	✓	✓		✓		✓			✓	

Willumsen (2006)	✓	✓	✓		✓		✓			


## Results

There was empirical evidence for seven of the 10 originally identified mechanisms: ‘Inspiring intent to work together’; ‘Creating the conditions to work together’; ‘Balancing multiple perspectives’; ‘Working with power’; ‘Taking a wider view’; ‘Commitment to learning and development’; and ‘Clarifying complexity’. There was insufficient evidence to identify two (‘Adaptability of leadership style’ and ‘Planning and coordinating’) as discrete mechanisms, therefore they were incorporated into the remaining seven mechanisms. There was no empirical evidence for the mechanism, ‘Fostering resilience’, although stakeholders felt this was potentially an important, long-term component of leadership. Key findings from the empirical evidence and stakeholder consultation are presented below. Examples of specific Context-Mechanism-Outcome configurations identified in the research papers are also presented below in text boxes:

### Mechanism 1: Inspiring intent to work together (n = 22)

#### Empirical evidence

Empirical evidence suggested it was important for leaders to *inspire* their team for integrated working by having a clear vision for collaboration and to articulate this vision with passion [23,24,25,26,27]. Successful leaders directed change according to this vision, rather than being ‘swept along’ by external events [23]. Several characteristics of ‘inspiring’ leaders were identified in the literature, including being visible [27,28]; able to gain the trust and respect of others [27,29,30,31]; a good communicator [23,24,26,27,32,33]; able to develop strong interpersonal relationships with colleagues [23,29,31]; and able to build a culture of interdependency, reciprocity and collaboration [24,34]. Leaders who openly recognised the time, effort, and skills that others contributed to integrated working made staff feel respected and motivated to contribute more [26]. Equally, conveying genuine respect for the views of all staff, regardless of affiliation or power, reinforced principles of inclusion and elevated members’ respect for leadership [26]. The importance of leader credibility and legitimacy was also identified [23,26,27,31,34], and was gained through having knowledge of both health and social care through direct experience of working in both fields [34]; being associated with previously successful developments [27]; or through one’s personality [23]. ***Box 1*** highlights a CMO configuration for the presence of this mechanism.

**Box 1: CMO configuration for the *presence* of the ‘Inspiring intent to work together’ mechanism [35]**

Staff within a newly integrated Mental Health NHS and Social Care Trust in the UK were conflicted around their expectations for the culture of the new Trust – they wanted to retain characteristics of their old health and social care cultures whilst also recognising the need for a new culture. This aspiration was acknowledged by the Chief Executive, who assured staff that the culture of the new Care Trust would maintain the best components of the previous organisations. This positive early experience of integration at a systems level formed a foundation for the importance of partnership working between health and social services, which became one of the core values of team level leaders within the Care Trust.

**The process of integrating services into a new Mental Health NHS and Social Care Trust means staff feel conflicted in their expectations for the culture of the new Trust (C)** → **The Chief Executive openly acknowledges and addresses any concerns that staff members have, including clarifying that the culture of the Care Trust will maintain the best components of their previous organisations (M+, Resource) and staff are reassured by this (M+, Reasoning)** → **The positive experience of integration at a systems level forms a foundation for the importance of partnership working between health and social services (O+) and becomes one of the core values of team level leaders within the Care Trust (O+)**.

#### Stakeholder feedback

Although identified as a vital component of leadership within the literature, stakeholders’ experience was that inspirational leadership alone was insufficient and needed to be supplemented by a range of other attributes:

“Whilst inspiration is important, other qualities are equally important… the leader has not only the inspirational skills but has also taken the time to do the work and find out what is involved on the ground.”

Furthermore, whilst developing a shared vision was identified as key within the literature, stakeholders noted that little emphasis was given to leaders creating this vision around the needs of the individual patient/service user. They also highlighted the need for a leader’s vision to be authentic, credible, and ethical if it was to be sustainable.

### Mechanism 2 – Creating the conditions to work together (n = 22)

#### Empirical evidence

Whilst the ‘Inspiring intent to work together’ mechanism referred to the need for leaders to have a clear vision for integrated working, which they could articulate clearly to others in an inspirational way, the ‘Creating the conditions to work together’ mechanism involved leaders ensuring that the appropriate systems were in place to enable this vision to be achieved. Little detail was provided for this mechanism, though some examples included setting and driving an agreed strategy [36]; re-drafting job descriptions to facilitate collaborative working [31]; or creating a framework for action [26] or ‘rules of engagement’ [37], to mobilise resources and guide action toward long-term aims [26,37]. Other examples involved leaders putting in place effective organisational systems and processes associated with governance, finances, human resources management and IT systems to accommodate service integration [24]. ***Box 2*** provides a CMO configuration for the absence of this mechanism.

**Box 2: CMO configuration for the *absence* of the ‘Creating the conditions’ mechanism [38]**

A Swedish case study of clinical integration efforts following a hospital merger described a team level leader’s belief that the new systems level leader focussed too much on a *“tough business management culture[…] which does not really fit the realities of a hospital”* and was overly concerned with reducing staff numbers. Because of this, the team level manager resigned from his position soon after his appointment, much to the disappointment of his team.

**Hospital merger requires the integration of pre-existing clinical departments and new leadership roles (C)** → **New systems level leader focussed on a ‘tough, business management culture’ concerned with reducing staff numbers (M-, Resource) but this did not fit with the vision of the team leader, who was concerned about job losses (M-, Reasoning)** → **Resignation of a popular, experienced clinical leader (O–) and disappointment from the team (O–)**.

#### Stakeholder feedback

Stakeholders agreed that inspiration alone was insufficient to effectively fulfil the requirements of the leadership role. However, it was suggested that the literature focussed excessively on the practical attributes of ‘creating the conditions’ and ignored the social skills also required. In their experience, ‘creating the conditions’ required leaders to have the emotional intelligence to create a culture of psychological safety, including developing an environment of transparency, openness and freedom to communicate without fear of repercussion. This required leaders to have self-awareness, motivation, and empathy. Consequently, one participant suggested that leaders of integrated care teams and systems were required to be more ‘evolved’ than their business world equivalents.

### Mechanism 3 – Balancing multiple perspectives (n = 20)

#### Empirical evidence

Historical power imbalances between health and social care mean the latter is often perceived as ‘the poor relation’ and integrating these services did not necessarily mean that social care would be given a higher political priority [39]. Leaders therefore needed to be mindful of this imbalance to try and prevent this historic pattern recurring [39]. Other key skills of leaders were identified as the ability to encourage team members to appreciate the core skills and expertise of others, to bridge diverse cultures, to manage difficult conversations and remove any obstacles to change [31,33,34,36,40,41,42,43]. Integrated working could be difficult due to differences in professional language, attitude and values [30] but successful leaders were able to consider the circumstances and ways of thinking of different disciplines and balance them accordingly [28,32]. Team members trusted that they could call upon their leaders to help resolve conflict amongst staff and leaders were able to do so effectively [24,30,32,43,44]. ***Box 3*** provides a CMO configuration for the presence of this mechanism.

**Box 3: CMO configuration for the *presence* of the ‘Balancing multiple perspectives’ mechanism [30]**

A Swedish study exploring collaboration between welfare agencies in the field of vocational rehabilitation reported that team leaders deliberately involve themselves in the steering committees or working groups of different collaborative projects to reduce territorial behaviour amongst staff and overcome differences in professional languages, attitudes and values. As a consequence, team leaders have to wear *“two different hats”* – both as a leader of their team and as a steering group member for collaborative projects and it can be difficult for them to balance loyalties to both groups.

**Team leaders are given time to develop integrated working and learn about different competencies within the team (C)** → **Team leaders deliberately involve themselves as members of steering committees and working groups for various collaborative projects (M+ Resource) and their presence makes staff reduce their territorial behaviours (M+, Reasoning)** → **Team leaders experience difficulties balancing loyalties to different groups (O–)**.

#### Stakeholder feedback

Whilst research generally identified a leader’s role was to resolve conflict, stakeholders’ experiences were more diverse and there was limited consensus over the way in which leaders should respond to it. Some suggested that conflict should be actively and intelligently resolved by leadership, but others felt it was more productive for conflict to be ‘held’ by leaders, as they challenged their team to work out problems themselves. These stakeholders considered a willingness to have difficult conversations with colleagues across different organisations and specialisms was evidence of a leaders’ attempts to deal with conflict, without attempting to resolve it directly.

### Mechanism 4 – Working with power (n = 16)

#### Empirical evidence

There was empirical evidence of the fundamental importance of leaders having a requisite level of power (including authority, influence, and responsibility) in order to support the process of integration [28,37]. Leaders also required the capacity for change, including leaders who were willing to adjust their role [31] and had the ability to adjust their leadership style, driving an agenda forward when required but letting it go when necessary [25]. By sharing power to set priorities, allocate resources and evaluate performance, leaders fostered a sense of joint ownership and collective responsibility thereby increasing the system’s effectiveness [26]. Researchers suggested that authoritative, directive and controlling leadership styles were generally inappropriate, whereas shared and distributive leadership approaches were likely to be more productive [34,45] and lead to greater team synergy [33]. However, on occasion, hierarchical leadership was deemed to be necessary or inevitable, particularly when dealing with legal or policy challenges [45] or when tackling performance [34]. ***Box 4*** highlights a CMO configuration for the absence of this mechanism.

**Box 4: CMO configuration for the *absence* of the ‘Working with power’ mechanism [38]**

Following a merger of two hospital departments in Sweden, the newly employed team leader refused to relinquish control to the team members over decisions around the structure of their new department. After asking for his team’s suggestions, the leader overrode their proposals with his own, more ambitious plan. This move upset many team members, particularly the physicians, and led to feelings of mistrust and suspicion toward the leader. From then on, many physicians no longer attended collaboration meetings for the merger.

**Hospital merger requires the integration of pre-existing clinical departments and decisions need to be made around the structure of the new department (C)** → **Team leader asks for the team’s suggestions about how to structure the department (M+, Resource) but overrides their proposals with their own, more ambitious plan that the team think has nothing to do with their work (M-, Reasoning)** → **This upsets team members (O–) and many physicians do not attend further collaboration meetings (O–)**.

#### Stakeholder feedback

Stakeholders felt that power dynamics in integrated care teams were more nuanced and sophisticated than the empirical evidence suggested. Power was described as working positively and negatively, with power moving through the system, rather than operating from top to bottom. Stakeholders also discussed the notion of ‘borrowed’ power, whereby permission or advocacy of somebody with decision-making power could be productive when distributed evenly throughout an integrated team. Stakeholders additionally referred to the absence of the service user voice throughout the research literature and the lack of power patients/service users held within integrated care teams and systems. Given that a primary aim of integrated care is to develop a patient/user-centred approach, the absence of the patient/service user voice and their relative lack of power are concerning.

### Mechanism 5 – Commitment to learning and development (n = 14)

#### Empirical evidence

Empirical evidence stated that an important component of integrated working was having the opportunity to share experiences and learn from others [23]. It was important for team leaders to both inspire learning within their teams and to show commitment to learning themselves. There was also a requirement for integrated teams to be willing and flexible to change and evolve according to need [36,37,46,47,48] but this was more difficult for organisations accustomed to ‘silo-working’ or where funding models supported care in silos [36,46]. An overarching context for this mechanism was the presence of an organisational culture that demonstrated mutual respect and understanding, which was crucial for nurturing learning and innovation within organisations [36]. Interprofessional and interorganisational training and education programmes were also described as helping teams to break down misconceptions and support integrated working [23,36,41,46]. ***Box 5*** provides a CMO configuration for the absence of this mechanism.

**Box 5: CMO configuration for the *absence* of the ‘Learning and innovation’ mechanism [36]**

An Australian case study contrasting two contexts of healthcare governance found it imperative that Board level leaders recognised the need for innovation and supported it as a key strategy for integrated working. However, there were reports of a focus on short-term political gains rather than long-term solutions and examples of ‘centralised bureaucratic control’ which resulted in a culture of risk aversion at Board level and which reduced teams’ ability to innovate, even when individual members were willing and capable of doing so.

**‘Centralised bureaucratic control’ at Board level (C)** → **Systems leaders become risk averse (M-, Resource) and so focus on short-term political gains rather than long-term solutions (M-, Reasoning)** → **Team members’ ability to innovate are limited, even when individual members are willing and capable of doing so (O–)**.

#### Stakeholder feedback

Whilst the original definition of the mechanism talked of the need for leaders to be able to think outside the box and to ‘fail well’, stakeholders believed leaders were often not given the opportunity to fail, due to the rigours of the systems in which they operate. There was consensus that current environments would benefit from a cultural shift, in which individuals felt comfortable to both feed their curiosity and develop in a way which accepted the inevitability of mistakes. The tension between innovation and the practical constraints of working within integrated care was also highlighted. There was suggestion that effective leaders needed to understand the gap between their own visions and the reality of working within integrated care to understand the experiences of their team:

“… if you don’t feel like you can disclose what your real world is like, then they won’t hear what that real world is like. So it is tied in with the cultural implications of psychologically safe environment and the ability of the leader to just sit and simply listen, so the staff don’t fear the repercussions of actually telling them things without punishment”.

### Mechanism 6 – Taking a wider view (n = 13)

#### Empirical evidence

Empirical evidence suggested it was important for leaders to have a deep, intuitive sense of how their community worked and its needs [24,26,31]. Leaders should look beyond the interests of individual organisations and even the interests of the integrated system and focus on higher order cause, effect and prevention, rather than on symptoms or quick fixes [26]. To achieve this, they needed strong, pre-existing networks [24] and to demonstrate a willingness to work with and learn from a range of different individuals and organisations [26,31,48]. Leaders needed to be skilled in navigating through complex and sensitive political issues [24] and to know who were the ‘right’ people to engage with at a strategic level in order to achieve change [25]. ***Box 6*** highlights a CMO configuration for the presence of this mechanism.

**Box 6: CMO configuration for the *presence* of the ‘Taking a wider view’ mechanism [37]**

A clinical merger of two hospital sites in the US took place with executive leaders at both sites responsible for designing and implementing the vision and future of the newly merged entity. It was understood from the beginning that for the merger to succeed, these leaders had to be willing to put aside personal interests in order to convince the rest of the organisations that the integrated healthcare system would be a better option for all parties. They achieved this through, *“communication, compromise and time in getting to know one another”* (p676) so that trust was developed between the executive staff and the senior administrators and clinical leaders at both hospital sites.

**Clinical merger of two hospital sites with executive leaders at both sites responsible for designing and implementing the future of the newly merged entity (C)** → **Both leaders appreciate that for the merger to succeed, they have to put aside personal interests and convince the rest of the organisations that the integrated healthcare system would be a better option for all parties (M+, Resource). They achieve this through regular communications as a whole group whilst simultaneously keeping local site meetings to a minimum. They also ensure no programme would be moved from one site to another without considering the impact of this (M+, Reasoning)** → **This instils trust amongst all team members (O+)**.

#### Stakeholder feedback

Whilst empirical evidence highlighted the importance of leaders knowing who the ‘right people’ were to engage with at a strategic level, stakeholders felt this could result in leaders seeking connections with *“like-minded”* individuals, creating a bias in outlook. It was suggested that associating only with like-minded individuals was reflective of anxiety, which may have been caused by (or contributed to) the disruptive and unsettling capacity of integrated care. It was also commented that working only with like-minded professionals encouraged the continuity of conventional assumptions around leadership and raised questions about the diversity of integrated care.

### Mechanism 7 – Clarifying complexity (n = 10)

#### Empirical evidence

There was empirical evidence of the importance of leaders being clear about the management structure, the contributions required from all participants in the system and the rules governing how the partnership should work, as staff felt unprepared and de-motivated when there was an absence of clear and consistent communication about what was required [46]. There was also evidence that leaders needed the ability to navigate through complex political landscapes and possess the relevant skills to oversee and manage complex clinical governance frameworks and practices, workgroup structures, and financial systems [24]. This included the ability to introduce changes in a controlled manner, so teams were not overwhelmed by change [48]. ***Box 7*** provides a CMO configuration for the absence of this mechanism.

**Box 7: CMO configuration for the *absence* of the ‘Clarifying complexity’ mechanism [38]**

A merger of two departments from Swedish hospitals caused a lack of simplification and clarity over leadership and reporting lines at a systems level. This left staff members feeling frustrated and confused and was time consuming, as staff then needed to report to several managers.

**Two clinical departments combine as part of a hospital merger (C)** → **Systems leaders instigate a complex management reporting structure, which means that staff have to report to more leaders than before (M-, Resource) and staff fail to understand the reasoning behind this new structure (M-, Reasoning)** → **This leaves staff feeling confused and frustrated (O–)**.

#### Stakeholder feedback

Although there was little empirical evidence for this mechanism, stakeholders maintained that it was an important responsibility of integrated care leaders to navigate the tension between certainty and uncertainty and to translate this to the team. It was commented that policy documents, such as the NHS Long Term Plan [2], did little to address the day-to-day complexities of working in an integrated system and that there was no leadership blueprint in these settings. Furthermore, the empirical evidence implied that the way in which a leader responded to complexity and how this impacted upon their decision-making were indicative of their perceived effectiveness. Yet stakeholders felt the way in which a leader talked to people during the decision-making process was the critical criterion for success and not necessarily the result of those decisions.

### Mechanisms 8 and 9 – Planning and coordinating (n = 5) and Adaptability of leadership style (n = 3)

Although five research papers discussed the ‘Planning and coordinating’ mechanism [24,31,32,43,49] and three discussed ‘Adaptability of leadership style’ [25,26,31], the findings from these papers did not suggest these were discrete mechanisms. Instead, these findings were incorporated into the other seven mechanisms.

### Mechanism 10 – Fostering resilience (n = 0)

#### Empirical evidence

No research papers were found to have discussed this mechanism.

#### Stakeholder feedback

Stakeholders were unsurprised that no research evidence emerged to support the initial programme theory but did have comments to make. They felt that no amount of resilience could help leaders of integrated systems with complex issues. Some considered that the term ‘resilience’ was code for ignoring/managing anxiety, without understanding why anxiety had been evoked. They therefore felt that successful leaders were those with an understanding of the necessity, practicality, political nature, and intense psychological impact of providing integrated care leadership. The longitudinal nature of developing effective integrated care systems was also highlighted, with stakeholders noting that it was a non-linear process, and that resilience-building was a long-term endeavour.

## Discussion

This research offers timely and unique perspectives on the mechanisms of leading integrated care. A key finding was that there was little evidence specifically addressing leadership of integrated care teams and systems, despite the widespread policy rhetoric and partial implementation of this model of organising services. There are several potential reasons for this. Although collaboration in health and social care has been part of government policy in England since the 1990s, the integration of health and social care takes the practice of collaboration further and deeper into organisations. This means that research has not been undertaken or completed. The limited research that does exist, however, focuses on the implementation and outcome of service innovation rather than on its structural underpinnings or assumes that leadership is a homogenous activity that is transferable across different settings and therefore not a research priority. It is also important to note the scant evidence of the contexts that influenced how leaders work and even less evidence on the outcomes. This made it challenging to link mechanisms to specific contexts and associated outcomes, which limited the degree to which we could draw definitive conclusions about what works, for whom in what circumstances. However, making explicit some of the assumptions about how leaders lead integrated care teams and systems has provided new perspectives. These were interrogated and challenged by our stakeholder group, offering new insights and fresh theoretical grounding that can be built upon, developed, and tested further. Thus, despite the limited research available in this field, we have been able to develop programme theories which explore contexts, reasoning, resources and outcomes. These have been highlighted in Table 3 (see Supplementary material).

The review also identified a lack of practical guidance about how to lead within integrated care. Throughout the evidence, there were general statements of the important activities that leaders do in leading integrated care, yet there was little explanation about how leaders undertook these activities, their reasoning of what the best approach would be, the trade-offs made, and the challenges encountered. Policy documents were thought to do little to address the complexities of working in an integrated system and our stakeholders felt there was no blueprint for leadership in integrated care. This further limits our understanding of what aspects of leadership work, for whom and in what circumstances. As our stakeholders highlighted, an important component additionally absent from the review was evidence of the patient/service user perspective. A central policy driver for the introduction of integrated care systems was the need for services to be integrated around the patient, to provide the best patient experience and the best value for money. It is a stark finding that we found no evidence of the patient/service user perspective of leadership or involvement in leadership of integrated care teams and systems.

The strongest evidence found in the review was around how leaders inspire people’s intent to work together within integrated care. This evidence focused on who the leader *is* rather than what the leader *does* and referred to their personality, characteristics, and ability to inspire other people. Yet stakeholders were clear that inspiration alone was not enough – a leader must also be knowledgeable, skilled and spend time on finding out what is involved on the ground. Stakeholders referred to aviation safety, stating, “you don’t really care who the pilot is and how inspirational the pilot is when you get on the aeroplane, you just want them to fly the plane safely.” This overemphasis on the influence of individuals is recognised in leadership theory. The culture of individual leaders as ‘heroes’, romanticising their individual abilities and dispositions, is thought to prioritise the importance of being in control and having the power to decide, steer and influence others. This overestimates a leaders’ contribution and influence [50,51] and can obscure, in part, the tensions and complexities inherent in leadership [52]. An alternative perspective is processual leadership [53], which views leadership as an ongoing process of social interaction and negotiation with all members of an organisation who participate in it and influence the organisation’s activity. Developing processual leadership practices that are attuned to a complex, changing, organisational context is thought to require recognition of the value of disagreement, tensions, and dissent. This is not to decry consensus but to acknowledge that it is not always achievable where stakeholders inevitably have different views and professional values. Leadership in organisations that are characterised by complexity and ambiguity, like integrated care systems, require a ‘viable sense of self-as-a-leader’ where leaders accept that social reality is constantly changing, and that control is an illusion [51]. However, maintaining a processual approach of leadership within an organisation or system, where the dominant conception of the ‘hero’ leader is deeply embedded and where leaders perceive high levels of ambiguity and insecurity, is extremely difficult [51]. We propose that perpetuating the importance of individual characteristics of leaders reduces the scope for integrated care teams and systems to develop leadership practices that are attuned to the complexity of multi-sectoral, multi-organisational and multi-professional working.

Finally, this review found the power and influence of leaders to be very important. Power was held by many people to varying degrees – by government, across the sectors, within organisations and by individuals at all levels of activity. Historic imbalances of power between health and social care were clear, as were higher levels of influence and power held by members of the medical profession. Stakeholders commented that the nature of power is far more complex and nuanced than the evidence suggests, therefore questions remain about how leaders of integrated care see their power and reason how to use it.

## Conclusion

To our knowledge, this is the first theory-informed realist review of leadership of integrated care teams and systems. It makes a significant contribution to the understanding of what is known and, perhaps more importantly, to the gaps in the empirical evidence. However, making explicit some of the assumptions about how leaders lead integrated care has provided new perspectives, that have been tested and refined from the perspective of a range of stakeholders who worked with us. This offers fresh theoretical grounding that can be built on, developed, and tested further.
